# Supplementary material for: The impact of the land-to-sea transition on evolutionary integration and modularity of the pinniped backbone
Source: Commun Biol. 2023 Nov 10;6:1141. doi: 10.1038/s42003-023-05512-8 (PMC10638317; doi:10.1038/s42003-023-05512-8)
Supplement: Supplementary file 2 — Supplementary information [file 42003_2023_5512_MOESM2_ESM.pdf]

# SUPPLEMENTARY METHODS

## CT-scanning

The material used in this section comes from the Zooparc de Beauval in France and the Oceanogràfic of Valencia. All the animals were anaesthetized and underwent a computerized tomography for routine matters of each center. The material used is: *Procyon lotor* ZPB_PL_003, and *Zalopus californianus* ZPB_ZC_004 *and Phoca vitulina* OV_PV_001.

The computed tomography at the Oceanographic institution of Valencia (Spain) were performed with Soamatom Volume Access (Siemens). At the French institution of Zooparc de Beauval, the specimens were scanned using a Philips Brillance 64 machine. Supplementary Data 19 shows the acquisition parameters of the tomographic process. The parameters used in each CT-scan for collecting the data are shown in Supplementary Data 19.

## Virtual dissection of the specimens

To obtain the surface area of the epaxial and hypaxial muscles at each slice we used the following protocol: (i) we applied enhancement filters to the stack of 16-bit TIFF images using ImageJ v.1.53t, (http://rsbweb.nih.gov/ij/)[1-3]. The resulting images are prepared for enhanced visualization of muscle components. In this case, the images are not converted to 8 bits as in previous works[3], and we used the 16 bits images for the correct visualization of muscles and other tissue components. The segmentation process was performed using the 3D slicer software v. 5.2.2 (www.slicer-org)[4]. In medial position within each vertebra, one slice per vertebra was segmented to collect the distribution of the epaxial and hypaxial muscles along the spine from C1 to the last lumbar, 27 slices in total. The segmentation data for the epaxial and hypaxial muscles were saved in DICOM format. These data were

imported into ImageJ. In both cases, all images were normalized at 0.5% and the histogram was adjusted to the range of interest (ROI) using the semiautomatic brightness/contrast tool of ImageJ[1,2]. Applying the *thresholding tool* to select the areas of interest and the binary tool, we convert the stack of images into binary images. The last step is to calculate the areas of each slice automatically with the *Analyze particles tool* from the Analyze menu in ImageJ[1,2]. The results obtained for the areas in mm2 of the epaxial and hypaxial muscles are shown in Supplementary Data 20-22.

## Supplementary figures

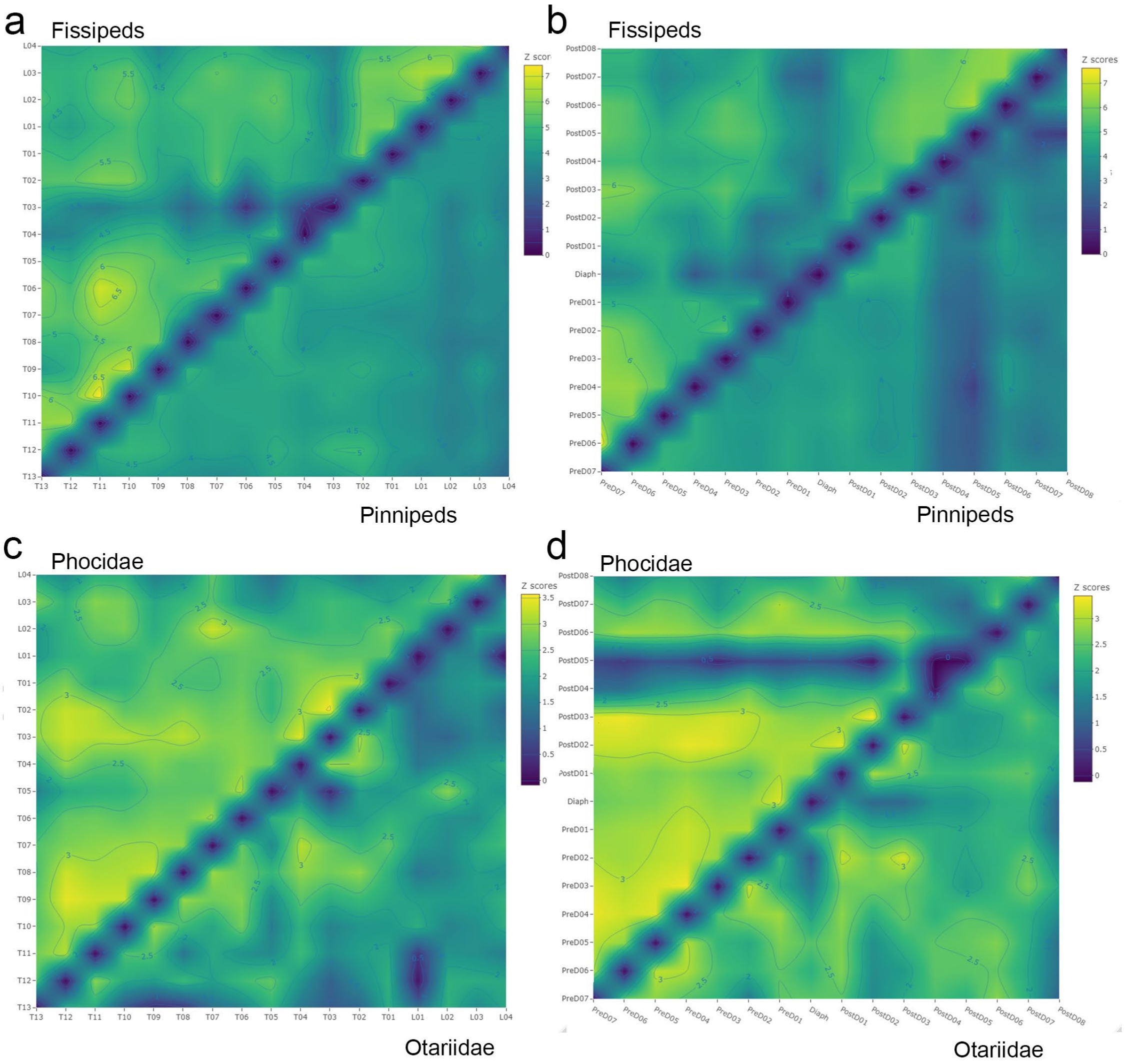


**Figure S1.** Heatmaps showing the strength of integration between each pair of presacral vertebrae. (A) strength of integration of fissipeds and crown pinnipeds using the "thoracolumbar boundary count" count procedure. The upper semimatrix corresponds to the Z-scores of fissipeds, all taken from ref.16 and the lower semimatrix to the Z-scores

of pinnipeds obtained in this study. (B) strength of integration of fissipeds and pinnipeds using the "Diaphragmatic start" count procedure. The upper semimatrix corresponds to the Z-scores of fissipeds taken from Martín-Serra et al.[16], and the lower semimatrix to the Z-scores of pinnipeds obtained in this study. (C) strength of integration of phocids and otariids using the "thoracolumbar boundary count r" count procedure. Upper and lower semi-matrixes correspond to Z-values of phocids and otariids, respectively. (D) strength of integration of phocids and otariids using the "Diaphragmatic start" count procedure. Upper and lower semi-matrixes correspond to Z-values of phocids and otariids, respectively.

## Supplementary tables and references

**Table S1**. Specimens included in the study. Museum abbreviation: LACM, Natural History Museum of Los Angeles County (LA, USA); MNHN, Muséum National d'Histoire Naturelle (Paris, France); NHMB, Natural History Museum of Basel (Basel, Switzerland); ZMUC, Zoological Museum of the University of Copenhagen (Copenhagen, Denmark); AMNH, American Museum of Natural history; NMS, National Museum of Scotland.

| **Species** | **Specimen ID** | **Family** | **Museum** |
|---|---|---|---|
| *Odobenus rosmarus* ♀ | 150 | Odobenidae | ZMUC |
| *Odobenus rosmarus* ♂ | n/a | Odobenidae | NHMB |
| *Arctocephalus australis* | 100341 | Otariidae | AMNH |
| *Arctocephalus galapagoensis* | 100319 | Otariidae | AMNH |
| *Arctocephalus gazella* ♀ | ZMUC 944 | Otariidae | ZMUC |
| *Arctocephalus gazella* | ZMUC 1081 | Otariidae | ZMUC |
| *Arctocephalus pusilus* | NA | Otariidae | AMNH |
| *Arctocephalus townsendi* | 76844 | Otariidae | AMNH |
| *Callorhinus ursinus* | AMNH 1878-198 | Otariidae | AMNH |
| *Eumatopias jubatus* | NA | Otariidae | MNHN |
| *Neophoca cinerea* ♂ | ZMUC 838 | Otariidae | ZMUC |
| *Otaria flavescens* | ZMUC 148 | Otariidae | ZMUC |
| *Otaria flavescens* ♂ | ZMUC 854 | Otariidae | ZMUC |
| *Zalophus californianus* | ZMUC 263 | Otariidae | ZMUC |
| *Zalophus californianus* | ZMUC 384 | Otariidae | ZMUC |
| *Zalophus wollebaeki* | 63957 | Otariidae | AMNH |
| *Cystophora cristata* ♂ | ZMUC 1134 | Phocidae | ZMUC |

| | | | |
|---|---|---|---|
| *Erignathus barbatus* | ZMUC 958 | Phocidae | ZMUC |
| *Erignathus barbatus* | ZMUC 809 | Phocidae | ZMUC |
| *Erignathus barbatus* | ZMUC 974 | Phocidae | ZMUC |
| *Halichoerus grypus* | ZMUC 416 | Phocidae | ZMUC |
| *Halichoerus grypus* | ZMUC 455 | Phocidae | ZMUC |
| *Halichoerus grypus* | ZMUC 975 | Phocidae | ZMUC |
| *Halichoerus grypus* ♂ | ZMUC 976 | Phocidae | ZMUC |
| *Histriophoca fasciata* | NA | Phocidae | AMNH |
| *Leptonychotes weddellii* | 34250 | Phocidae | AMNH |
| *Mirounga angustirostris* | ZMUC 839 | Phocidae | ZMUC |
| *Mirounga leonina* | ZMUC 957 | Phocidae | ZMUC |
| *Monachus monachus* | 73607 | Phocidae | AMNH |
| *Monachus schauinslandi* ♀ | NA | Phocidae | AMNH |
| *Monachus tropicalis* | 10431 | Phocidae | LACM |
| *Phoca groendlandica* ♂ | ZMUC 780 | Phocidae | ZMUC |
| *Phoca groendlandica* ♂ | ZMUC 782 | Phocidae | ZMUC |
| *Phoca groendlandica* ♂ | ZMUC 961 | Phocidae | ZMUC |
| *Phoca largha* | 15817 | Phocidae | AMNH |
| *Phoca vitulina* | M180/02 | Phocidae | NMS |
| *Phoca vitulina* | ZMUC 160 | Phocidae | ZMUC |
| *Phoca vitulina* | ZMUC 1077 | Phocidae | ZMUC |
| *Pusa hispida* | ZMUC 157 | Phocidae | ZMUC |
| *Pusa hispida* | ZMUC 783 | Phocidae | ZMUC |
| *Pusa hispida* | ZMUC 803 | Phocidae | ZMUC |
| *Pusa hispida* | ZMUC 950 | Phocidae | ZMUC |
| *Pusa sibirica* | 185595 | Phocidae | AMNH |

**Table S2**. Landmark descriptions.

| Landmark | Definition |
|---|---|
| **1** | Most dorsal point of the spinous process in the cranial side |
| **2** | Base of the spinous process in the cranial side |
| **3** | Mid-point of the neural arch in the cranial side |
| **4** | Right pre-zygapophysis most caudal point |
| **5** | Right pre-zygapophysis, most cranial point |
| **6** | Right pre-zygapophysis, most lateral-mid point, |
| **7** | Right pre-zygapophysis, most medial-mid point |
| **8** | Left pre-zygapophysis, most caudal point |
| **9** | Left pre-zygapophysis, most cranial point |
| **10** | Left pre-zygapophysis, most lateral-mid point |
| **11** | Left pre-zygapophysis, most medial-mid point |
| **12** | Dorsal point of the cranial facet of the vertebra body |
| **13** | Ventral point of the cranial facet of the vertebra body |
| **14** | Right-middle point of the cranial facet of the vertebra body |
| **15** | Left-middle point of the cranial facet of the vertebra body |
| **16** | Cranial point, right transverse process |
| **17** | Cranial point, left transverse process |
| **18** | Most dorsal point of the spinous process in the caudal side |
| **19** | Base of the spinous process in the caudal side |
| **20** | Mid-point of the neural arch in the caudal side |
| **21** | Left post-zygapophysis, most cranial point |
| **22** | Left post-zygapophysis, most caudal point |
| **23** | Left post-zygapophysis, most lateral-mid point |
| **24** | Left post-zygapophysis, most medial-mid point |
| **25** | Right post-zygapophysis, most cranial point |
| **26** | Right post-zygapophysis, most caudal point |
| **27** | Right post-zygapophysis, most lateral-mid point |

| | |
|---|---|
| **28** | Right post-zygapophysis, most medial-mid point |
| **29** | Dorsal point of the caudal facet of the vertebra body |
| **30** | Ventral point of the caudal facet of the vertebra body |
| **31** | Left-middle point of the caudal facet of the vertebra body |
| **32** | Right-middle point of the caudal facet of the vertebra body |
| **33** | Caudal point on the left transverse process |
| **34** | Caudal point on the right transverse process |
| **35** | Cranial right point on the ventral crest |
| **36** | Cranial left point on the ventral crest |
| **37** | Cranial middle point on the ventral crest |
| **38** | Caudal right point on the ventral crest |
| **39** | Caudal left point on the ventral crest |
| **40** | Caudal middle point on the ventral crest |

## Supplementary references

1. Schneider, C.A., Rasband, W.S. & Eliceiri, K.W. NIH Image to ImageJ: 25 325 years of image analysis. Nat. methods. **9**, 671-675 (2012).

2. Pérez-Ramos, A. & Figueirido, B. Toward an "Ancient" virtual World: improvement methods on X-ray CT data processing and virtual reconstruction of fossil skulls. *Front. Earth. Sci.* **8**, 345 (2020).

3. Figueirido, B., Lautenschlager, S., Pérez-Ramos, A. & Van Valkenburgh, B. Distinct predatory behaviors in scimitar-and dirk-toothed sabertooth cats. *Curr. Biol.* **28**, 3260-3266 (2018).

4. Kikinis, R., Pieper, S.D. & Vosburgh, K.G. 3D Slicer: a platform for subject327 specific image analysis, visualization, and clinical support. Intraoperative imaging and 328 image-guided therapy (ed, Jolesz FA) 277-289 (Springer New York, 2014).
