# Supplementary material for: The impact of the land-to-sea transition on evolutionary integration and modularity of the pinniped backbone
Source: Commun Biol. 2023 Nov 10;6:1141. doi: 10.1038/s42003-023-05512-8 (PMC10638317; doi:10.1038/s42003-023-05512-8)
Supplement: Supplementary file 3 — Description of Additional Supplementary Files [file 42003_2023_5512_MOESM3_ESM.pdf]

Description of Additional Supplementary Files

File name: Supplementary Data 1
Description: Raw coordinates (x,y,z) of the landmarks digitized in each vertebra.

File name: Supplementary Data 2
Description: Results of Procrustes ANOVAs for all pinnipeds for the cervical plus joined thoracolumbar count procedure.

File name: Supplementary Data 3
Description: Results of Procrustes ANOVAs for all pinnipeds for the thoracolumbar boundary count procedure.

File name: Supplementary Data 4
Description: Results of Procrustes ANOVAs for all pinnipeds for the diaphragmatic start count procedure.

File name: Supplementary Data 5
Description: Results of Procrustes ANOVAs for all pinnipeds for the selected vertebrae procedure.

File name: Supplementary Data 6
Description: Results (p-values) obtained from phylogenetic and non-phylogenetic 2B-PLSs for each pair of vertebrae for the cervical plus joined thoracolumbar count procedure.

File name: Supplementary Data 7
Description: Results (p-values) obtained from phylogenetic and non-phylogenetic 2B-PLSs for each pair of vertebrae for the thoracolumbar boundary count procedure.

File name: Supplementary Data 8
Description: Results (p-values) obtained from phylogenetic and non-phylogenetic 2B-PLSs for each pair of vertebrae for the diaphragmatic start count procedure.

File name: Supplementary Data 9
Description: Results (p-values) obtained from phylogenetic and non-phylogenetic 2B-PLSs for each pair of vertebrae for the selected vertebrae procedure.

File name: Supplementary Data 10
Description: Results of Procrustes ANOVAs for phocids for the cervical plus joined thoracolumbar count procedure.

File name: Supplementary Data 11
Description: Results of Procrustes ANOVAs for phocids for the thoracolumbar boundary count procedure.

File name: Supplementary Data 12
Description: Results of Procrustes ANOVAs for phocids for the diaphragmatic start count procedure.

File name: Supplementary Data 13
Description: Results of Procrustes ANOVAs for phocids for the selected vertebrae procedure.

File name: Supplementary Data 14
Description: Results of Procrustes ANOVAs for otariids for the cervical plus joined thoracolumbar count procedure.

File name: Supplementary Data 15
Description: Results of Procrustes ANOVAs for otariids for the thoracolumbar boundary count procedure.

File name: Supplementary Data 16
Description: Results of Procrustes ANOVAs for otariids for the diaphragmatic start count procedure.

File name: Supplementary Data 17
Description: Results of Procrustes ANOVAs for otariids for the selected vertebrae procedure.

File name: Supplementary Data 18
Description: Pinniped Zscore values using the same landmark as in fissipeds [16].

File name: Supplementary Data 19
Description: Parameters and data acquisition of the study samples.

File name: Supplementary Data 20
Description: Surface areas of epaxial muscles (in mm2) for each slice corresponding to each vertebra, from C1 to L5.

File name: Supplementary Data 21
Description: Surface areas of hypaxial muscles (in mm2) for each slice corresponding to each vertebra, from C1 to L5.

File name: Supplementary Data 22

Description: Ratio values from dividing the Epaxial with Hypaxial area for each slice corresponding to each vertebra, from C1 to L5.
