# Supplementary material for: The impact of the land-to-sea transition on evolutionary integration and modularity of the pinniped backbone
Source: Commun Biol. 2023 Nov 10;6:1141. doi: 10.1038/s42003-023-05512-8 (PMC10638317; doi:10.1038/s42003-023-05512-8)
Supplement: Supplementary file 6 — Reporting Summary [file 42003_2023_5512_MOESM6_ESM.pdf]

nature portfolio

Corresponding author(s): Juan Miguel Esteban

Last updated by author(s): October, 17, 2023

Reporting Summary

Nature Portfolio wishes to improve the reproducibility of the work that we publish. This form provides structure for consistency and transparency in reporting. For further information on Nature Portfolio policies, see our Editorial Policies and the Editorial Policy Checklist.

Statistics

For all statistical analyses, confirm that the following items are present in the figure legend, table legend, main text, or Methods section.

n/a	Confirmed	
☐	☒	The exact sample size (*n*) for each experimental group/condition, given as a discrete number and unit of measurement
☐	☒	A statement on whether measurements were taken from distinct samples or whether the same sample was measured repeatedly
☐	☒	The statistical test(s) used AND whether they are one- or two-sided<br>*Only common tests should be described solely by name; describe more complex techniques in the Methods section.*
☐	☒	A description of all covariates tested
☒	☐	A description of any assumptions or corrections, such as tests of normality and adjustment for multiple comparisons
☐	☒	A full description of the statistical parameters including central tendency (e.g. means) or other basic estimates (e.g. regression coefficient) AND variation (e.g. standard deviation) or associated estimates of uncertainty (e.g. confidence intervals)
☐	☒	For null hypothesis testing, the test statistic (e.g. *F*, *t*, *r*) with confidence intervals, effect sizes, degrees of freedom and *P* value noted<br>*Give P values as exact values whenever suitable.*
☒	☐	For Bayesian analysis, information on the choice of priors and Markov chain Monte Carlo settings
☐	☒	For hierarchical and complex designs, identification of the appropriate level for tests and full reporting of outcomes
☐	☒	Estimates of effect sizes (e.g. Cohen's *d*, Pearson's *r*), indicating how they were calculated

Our web collection on statistics for biologists contains articles on many of the points above.

Software and code

Policy information about availability of computer code

Data collection	The landmarks in the 3D models were collected with the software Stratovan Check Point
Data analysis	-Geomorph package of R. version 3.1.0. Adams, D. C., M. L. Collyer, and A. Kaliontzopoulou. (2019). Geomorph: Software far geometric morphometric analyses. R package version 3.1.0. https://cran.r-project.org/package=geomorph.<br>- 3D models were edited with Meshlab. Cignoni, P., Callieri, M., Corsini, M., Dellepiane, M., Ganovelli, F., & Ranzuglia, G. (2008, July). Meshlab: an open-source mesh processing tool. In Eurographics ltalian chapter conference (Vol. 2008, pp. 129-136).<br>- Ape package of R. Paradis, E. & Schliep, K. Ape 5.0: an environment far modern phylogenetics and evolutionary analyses in R. Bioinfarmatics 35, 526-528 (2019).<br>- CT scans were segmented with 3D slicer software v. 5.2.2 (www.slicer-org). Kikinis, R., Pieper, S.D. & Vosburgh, K.G. 3D Slicer: a platfarm far subject specific image analysis, visualization, and clinical support. lntraoperative imaging and image-guided therapy (ed, Jolesz FA) 277-289.<br>- CT scanned specimens were analysed with ImageJ. Schneider, C.A., Rasband, W.S. & Eliceiri, K.W. NIH lmage to lmageJ: 25 325 years158 of image analysis. Nat. methods. 9, 671-675 (2012).

For manuscripts utilizing custom algorithms or software that are central to the research but not yet described in published literature, software must be made available to editors and reviewers. We strongly encourage code deposition in a community repository (e.g. GitHub). See the Nature Portfolio guidelines for submitting code & software for further information.

Data

Policy information about availability of data

All manuscripts must include a data availability statement. This statement should provide the following information, where applicable:

- Accession codes, unique identifiers, or web links for publicly available datasets
- A description of any restrictions on data availability
- For clinical datasets or third party data, please ensure that the statement adheres to our policy

The data is available in the Supplementary information as "Dataset1"

Human research participants

Policy information about studies involving human research participants and Sex and Gender in Research.

Reporting on sex and gender	This study does not involve human participants, their data or any derived biological material
Population characteristics	This study does not involve human participants, their data or any derived biological material
Recruitment	This study does not involve human participants, their data or any derived biological material
Ethics oversight	This study does not involve human participants, their data or any derived biological material

Note that full information on the approval of the study protocol must also be provided in the manuscript.

Field-specific reporting

Please select the one below that is the best fit for your research. If you are not sure, read the appropriate sections before making your selection.

☐ Life sciences ☐ Behavioural & social sciences ☒ Ecological, evolutionary & environmental sciences

For a reference copy of the document with all sections, see nature.com/documents/nr-reporting-summary-flat.pdf

Ecological, evolutionary & environmental sciences study design

All studies must disclose on these points even when the disclosure is negative.

Study description	he paper investigates the relationship between vertebral morphology and patterns of integration/modularity across the vertebral column of pinniped carnivorans. We also relate qualitatively how these patterns are related to the new functional demands imposed by the aquatic environment.
Research sample	We scanned 1075 presacral vertebrae belonging to the following specimens/species: (Species/Specimen ID/Family/Museum): Odobenus rosmarus, 150, Odobenidae, ZMUC; Odobenus rosmarus, n/a, Odobenidae, NHMB; Arctocephalus australis, 100341, Otariidae, AMNH; Arctocephalus galapagoensis, 100319, Otariidae, AMNH; Arctocephalus gazella, 944, Otariidae, ZMUC; Arctocephalus gazella , 1081, Otariidae, ZMUC; Arctocephalus pusilus, NA, Otariidae, AMNH; Arctocephalus townsendi, 76844, Otariidae, AMNH; Callorhinus ursinus, 1878-198, Otariidae, AMNH; Eumatopias jubatus, NA, Otariidae, MNHN; Neophoca cinérea, 838, Otariidae, ZMUC; Otaria flavescens, 148, Otariidae, ZMUC; Otaria flavescens, 854, Otariidae, ZMUC; Zalophus californianus, 263, Otariidae, ZMUC; Zalophus californianus, 384, Otariidae, ZMUC; Zalophus wollebaeki, 63957, Otariidae, AMNH; Cystophora cristata, 1134, Phocidae, ZMUC; Erignathus barbatus, 958, Phocidae, ZMUC; Erignathus barbatus, 809, Phocidae, ZMUC; Erignathus barbatus, 974, Phocidae, ZMUC; Halichoerus grypus, 416, Phocidae, ZMUC; Halichoerus grypus, 455, Phocidae, ZMUC; Halichoerus grypus, 975, Phocidae, ZMUC; Halichoerus grypus, 976, Phocidae, ZMUC; Histriophoca fasciata, NA, Phocidae, AMNH; Hydrurga leptonyx, 1970-325, Phocidae, AMNH; Leptonychotes weddellii, 34250, Phocidae, AMNH; Mirounga angustirostris, 839, Phocidae ZMUC; Mirounga leonina, 957, Phocidae, ZMUC; Monachus monachus, 73607, Phocidae, AMNH; Monachus schauinslandi, NA, Phocidae, AMNH; Monachus tropicalis, 10431, Phocidae, LACM; Phoca groendlandica, 780, Phocidae, ZMUC; Phoca groendlandica, 782, Phocidae, ZMUC; Phoca groendlandica, 961, Phocidae, ZMUC; Phoca larga, 15817, Phocidae, AMNH; Phoca vitulina, M180/02, Phocidae, NMS; Phoca vitulina, 160, Phocidae, ZMUC; Phoca vitulina, 1077, Phocidae, ZMUC; Pusa hispida, 157, Phocidae ZMUC; Pusa hispida, 783, Phocidae, ZMUC; Pusa hispida, 803, Phocidae, ZMUC; Pusa hispida,950, Phocidae, ZMUC; Pusa sibirica, 185595, Phocidae, AMNH.
Sampling strategy	No statistical methods were developed to predetermine sample size. We selected at least one column per species belonging to as much species as possible of the families Phocidae and Otariidae and the only living species of Odobenidae. We tried to cover a range of locomotor ecologies and body sizes, that are representative of the entire spectrum. The specimens included were based entirely upon availability in museum collections.
Data collection	The vertebrae were scanned in 3D with a Einscan Pro 2x Plus surface scanner. To capture the morphology of the vertebrae, we digitized 40 homologous landmarks on the cervical vertebrae (C03-C07), thoracic , and lumbar vertebrae. The landmarks were

	digitized with the software Stratovan Check Point and the x,y,z coordinates of each landmark were exported as a Text file. The data was collected by Juan Miguel Esteban.
Timing and spatial scale	The data was collected from 2021 to 2023
Data exclusions	No data were excluded from the analysis.
Reproducibility	We do not have experiments in our paper.
Randomization	Organisms were allocated into the three recognized families of living pinnipeds. This is specified in Table S1. This classification was relevant to investigate the association of vertebral integration/modularity in each of the species Phocidae and Otariidae.
Blinding	Blinding was not relevant in our study because we do not perform experimental procedures for data collection or analyses.

Did the study involve field work? ☐ Yes ☒ No

Reporting for specific materials, systems and methods

We require information from authors about some types of materials, experimental systems and methods used in many studies. Here, indicate whether each material, system or method listed is relevant to your study. If you are not sure if a list item applies to your research, read the appropriate section before selecting a response.

Materials & experimental systems

n/a	Involved in the study
☒	☐ Antibodies
☒	☐ Eukaryotic cell lines
☐	☒ Palaeontology and archaeology
☐	☒ Animals and other organisms
☒	☐ Clinical data
☒	☐ Dual use research of concern

Methods

n/a	Involved in the study
☒	☐ ChIP-seq
☒	☐ Flow cytometry
☒	☐ MRI-based neuroimaging

Palaeontology and Archaeology

Specimen provenance	ZMUC: Zoological Museum, University of Copenhaguen NHMB: Natural History Museum of Basel. AMNH: American Museum of Natural History LACM: Los Angeles County Museum
Specimen deposition	All the specimens are deposited in the collections aforementioned above.
Dating methods	No new dates are provided

☒ Tick this box to confirm that the raw and calibrated dates are available in the paper or in Supplementary Information.

Ethics oversight	No ethical was required as there is not dating methods.

Note that full information on the approval of the study protocol must also be provided in the manuscript.

Animals and other research organisms

Policy information about studies involving animals; ARRIVE guidelines recommended for reporting animal research, and Sex and Gender in Research

Laboratory animals	In this study, we used 3 CT-scanned animals that are in captivity in the Zooparc de Beauval in France and the Oceanografic of Valencia (Spain). All the animals were anaesthetized and underwent a computerized tomography for routine matters of each center. Therefore, the CT-scanns were not performed to the specific purposes of this study . The material used is: a Procyon lotor ZPB_PL_003 (male), a Zalopus californianus ZPB_ZC_004 (male) and a Phoca vitulina OV_PV_00l (female).
Wild animals	N/A
Reporting on sex	We do not perform sex-based analysis in this study beca use we are not interested in sexual dimorphism. In any case, as we do not performed CT-scans for the purpose of this study beca use we used previous CT-scanned specimens with available data at respective institutions, we just have access to the two males and one fema le.

Field-collected samples	N/A
Ethics oversight	COMITÉ DE CUIDADO y BIENESTAR ANIMAL DEL OCEANOGRÁFIC DE VALENCIA (SPAIN) INTERNAL WELFARE COMMITTEE OF THE ZOOPARC OF BEAUVAL

Note that full information on the approval of the study protocol must also be provided in the manuscript.
